# Image-Guided Radiation Therapy for Squamous Cell Cancer of the Head and Neck in a Specialized Peruvian Public Hospital

**DOI:** 10.7759/cureus.22569

**Published:** 2022-02-24

**Authors:** Lissett Jeanette Fernández-Rodríguez, María Alejandra Arens-Benites, Xavier Maldonado-Pijoan

**Affiliations:** 1 Department of Medicine, Hospital de Alta Complejidad Virgen de la Puerta, Trujillo, PER; 2 Radiotherapy Center, Hospital de Alta Complejidad Virgen de la Puerta, Trujillo, PER; 3 Radiation Oncology, Vall d'Hebron University Hospital, Barcelona, ESP

**Keywords:** cone-beam computed tomography, image guided radiotherapy, peru, intensity modulated radiotherapy, squamous cell cancer, head and neck cancer

## Abstract

Squamous cell cancer of the head and neck (SCCHN) often requires adjuvant radiotherapy. Radiotherapy for SCCHN is a challenge because the head and neck contain several critical organs that should receive minimal doses of radiation. These organs include the eyes, parotid glands, brainstem, spinal cord, mandible, and thyroid gland. Approaches like image-guided radiotherapy (IGRT) combined with volumetric modulated arc therapy hold the promise to focus radiation to the planning target volume and spare nearby structures while observing potential changes to patient anatomy during treatment to determine whether replanning is required. IGRT, however, requires the frequent imaging of patients to update the treatment plan. In this retrospective study, we present our findings of SCCHN patients treated in a public hospital in Peru. The patients reflected overall demographic trends associated with SCCHN. Each patient was imaged using computed tomography once before radiotherapy and once by cone-beam computed tomography (CBCT) during treatment, for a total of two images. Tumor displacement, planning target volume, gross tumor volume, and neck diameter were compared between the two images. Among the measurements, only a small statistically significant increase in gross tumor volume was observed between the images. However, a minority of patients did experience changes to anatomy, which highlights the need for continued research into criteria to determine which patients are likely to benefit from treatment replanning due to intra-treatment anatomical changes. Alternatively, a lack of frequent CBCT imaging before each session, due to high patient flows and limited staff resources, made it difficult to observe transient changes and trends in each patient. We conclude that the treatment and outcome improvements associated with IGRT are likely associated with frequent imaging during radiotherapy and properly selecting which patients will benefit most from this resource-intensive technique.

## Introduction

Each year, there are more than 500,000 new worldwide cases of squamous cell cancer of the head and neck (SCCHN) [1]. SCCHN is most frequently found in the oral cavity, tonsils, oropharynx, hypopharynx, larynx, and nasopharynx and is associated with tobacco exposure, alcohol dependence, and oncogenic viruses [2].

SCCHN can be treated in a variety of ways, including surgery, chemotherapy, immunotherapy, radiotherapy, or a combination of these, depending on the severity of the diagnosis. As with other cancers, treatment protocols should maximize tumor reduction, but not at the expense of healthy tissue [3]. Ideally, RT would deliver a high dose of ionizing radiation to the tumor, spare healthy tissue outside of the planning target volume, account for anatomical changes that take place during treatment, and maximize efficient use of equipment and specialist time. This is of particular challenge to radiotherapy (RT) because the head and neck contain several critically important organs, such as the eyes, parotid glands, brainstem, spinal cord, mandible, and thyroid gland.

Improved RT treatment approaches have followed two main branches. The first branch involves improving the precision of the radiation beam, as in intensity-modulated radiation therapy (IMRT) and volumetric modulated arc therapy (VMAT). However, when IMRT is used alone, it suffers from errors associated with the simulation process, positioning during treatment, and movement of the organs between sessions. This can cause a discrepancy between the expected and real radiation doses, which have been observed in multiple small studies [4-8]. Although it is an improved form of IMRT, VMAT likely suffers from the same limitations associated with patient positioning and relative movement of anatomical features [9].

The second branch, called image-guided radiotherapy (IGRT), involves using precision imaging at treatment sessions to eliminate errors in patient positioning and identifying patients that might need RT replanning due to changes in the size and relative position of the tumor [10,11]. In developed countries, such as in Europe, nearly all patients benefit from a combined IMRT/IGRT approach using recently-proposed best-practice recommendations [10]. IGRT usually involves taking a 2D or 3D cone-beam CT (CBCT) image before RT sessions to confirm patient, tumor, and organ at risk (OAR) positioning interspersed with RT replanning sessions if necessary. Using IGRT daily has allowed for the reduction of margin size and increasing fraction dose (hypofractionation) while maintaining local control [8,10]. This reduces the risk of patient toxicity. However, additional imaging with CBCT increases the ionizing radiation dose to patients and requires both instrument and trained specialist time. Therefore, it is not clear that all SCCHN patients would benefit from this approach.

Thus, searching for criteria that might differentiate which patient would benefit most from IGRT would be helpful. Additionally, nearly all studies of IGRT/VMAT have taken place in well-resourced hospitals with sufficient equipment to handle patient flows, while experiences in less than optimal situations are not as readily reported. Indeed, SCCHN is more lethal in developing countries because of high demand for services but a lack of infrastructure and expertise [12,13]. Therefore, we wish to share our experiences with IGRT/VMAT in a public Peruvian hospital.

## Materials and methods

Patients

The study population was patients with SCCHN diagnosed and treated at the Radiotherapy Center of the Hospital de Alta Complejidad Virgen de la Puerta, Trujillo, Peru, between February 2018 and March 2021. Inclusion criteria included a histological diagnosis of SCCHN, staged at T3-T4 N0 or T1-T4 N1-N3, age greater than 18 years, ECOG 1-2, and treatment using bilateral neck RT with IMRT with conventional fractionation and treatment planning occurring before treatment. Patients that had incomplete information in the Varian system or received previous RT treatment were excluded.

The study was approved by the Teaching, Research and Ethics Committee of the La Libertad Assistance Network of Training, Research and Teaching, approval code: PI N° 82 CIYE- O.C.I.Y D-RALL-ESSALUD-2021.

Initial imaging of tumors

Initial images were taken using helicoidal CT with an axial slice thickness of 2.5 to 5 mm using a Philips Brilliance 6 CT Scanner (Koninklijke Philips N.V., Amsterdam). Patients were in a supine position and immobilized using individualized thermoplastic masks that were later used for RT treatment. The image was taken approximately two weeks pre-treatment.

Definition of planning tumor volumes

The planning system Eclipse version 7.5 (Varian Oncology Systems) was used to delineate and calculate the dose distribution. A radiation oncologist manually contoured the limit of the tumor on each CT axial slice. Tumor volumes were defined according to International Commission on Radiation Units and Measurements Report 62 [14]. Gross tumor volume (GTV) included the primary tumor and affected lymph nodes. The CTV1 (high-risk clinical target volume) included the GTV and surrounding high-risk regions. The CTV2 (intermediate risk clinical target volume) included lower-risk nodal regions. To compensate for geometric uncertainty, such as the shape and movement of organs, 5 mm were added automatically to CTV to obtain planning tumor volumes (PTV1 & PTV2). The prescribed doses for PTV1 and PTV2 were 66 to 70 Gy and 51 to 54 Gy,, respectively.

Treatment planning parameters

The prescribed dose (D_pres_) must cover at least 95% of the target volumes, the near minimum dose must be greater than 93% of D_pres, and_ the near maximum dose (D_2%_) of the PTV should be less than 115% of D_pres_; PTV1 was considered the highest priority target volume. High priority limitations for normal critical structures were no more than 1.0 cm^3^ of the spinal cord could receive more than 48 Gy; no more than 1% of the brainstem could receive more than 54 Gy; D_2%_ for the mandible must be less than 70 Gy; the volume of parotid gland receiving more than 26 Gy must be less than 50% in at least one gland; D_2%_ of normal tissue should be less than D_pres_ [7].

Low priority constraints that should not compromise target coverage were mean absorbed dose (mean D) covering the lower mid-neck, oral cavity, and lips should be less than 40 Gy; D_2%_ for the eyes must be less than 50 Gy; D_2%_ for the cochlea must be less than D_pres_. Completed VMAT treatment plans were approved by a radiation oncologist.

Comparison of images

Patients were treated according to the plan using a Clinac 2300 model IX (Varian Medical Systems, Inc., USA, CL-IX-SO#320307634) linear accelerator. In the middle of the treatment, a cone-beam computed tomography (CBCT) image was taken of the patients using the same equipment to observe changes in the tumor and adjacent structures. The initial and mid-treatment images were overlaid and GTV, CTV, and PTV, tumor displacement, and coefficients of similarity were measured using the Varian system. Additionally, the diameter of the upper, middle, and lower neck was measured in both images. These measurements were compared to the initial CT image using a paired Student t-test.

## Results

A cohort of sequential patients treated at the radiotherapy center between February 2018 and March 2021 that met inclusion criteria was used for this study. The study group consisted of nine males and two females resident in Northern Peru, with an age of 59±12, range 46-85 years (Table 1). Tumor location was in the oropharynx (55%), oral cavity (27%), and larynx (18%). Clinical stages observed were IVA (46% of patients), IVB (18%), IVC (9%), II (18%), and III (9%). Cancers were treated with radiation (one patient), chemotherapy and radiation (five patients), radiation and biological therapy (two patients), or surgery, chemotherapy, and radiation (three patients). The majority of patients had ECOG scores of 0 or 1.

**Table 1:** Measured patient parameters. Initial measurements were taken before treatment using CT, while “mid” measurements were taken during treatment using CBCT. The only statistically significant difference observed between measurements was for GTV. The change is the difference between the initial and mid measurements.

#	Age (y)	Tumor location	Clinical stage	GTV, cm^3^			PTV1, cm^3^			PTV2, cm^3^			Upper Neck, cm			Middle Neck, cm			Lower Neck, cm		
				initial	mid	change	initial	mid	change	initial	mid	change	initial	mid	change	initial	mid	change	initial	mid	
1	85	larynx	T3NXMX	7.9	8.7	0.8	225.3	225.2	-0.1	113.8	111.8	-2	11.73	11.73	0	11.91	11.91	0	12.91	12.51	-0.4
2	69	tonsil	T4BN1M0	47.8	48.8	1	786.4	787.4	1	224.4	225.8	1.4	14.98	14.98	0	14.09	14.09	0	14.98	14.98	0
3	52	middle tongue	T2N0M0	33.5	34.6	1.1	817.4	817.6	0.2	141.2	142.5	1.3	10.95	10.95	0	10.63	10.63	0	13.12	13.12	0
4	69	larynx	T3NXMX	17.8	18.2	0.4	840.6	776.5	-64.1	61.2	62	0.8	12.87	12.87	0	11.97	11.97	0	11.84	11.84	0
5	54	tonsil	T2N1M0	31.6	31.9	0.3	147.7	148.2	0.5	131.5	132.5	1	14.1	14.1	0	14.48	14.48	0	16.72	15.44	-1.28
6	51	floor of mouth	T2N0M0	8	8.5	0.5	652.7	656.6	3.9	82	83.3	1.3	12.17	12.17	0	11.65	11.65	0	13.77	13.77	0
7	50	tonsil	T3N2M0	7.3	7.5	0.2	552.1	554.1	2	96.2	96.6	0.4	13.08	13.08	0	12.95	12.95	0	13.83	13.83	0
8	70	tonsil	T1N1M1	17.7	18.4	0.7	958.2	957.6	-0.6	180.1	180.7	0.6	15.15	15.15	0	14.2	14.2	0	15.22	15.22	0
9	47	middle tongue	T4BN1M0	57.9	57.9	0	701.9	659	-42.9	57.4	56.9	-0.5	13.46	13.46	0	12.37	12.37	0	14.47	14.47	0
10	59	tonsil	TXN1M0	9.2	9.1	-0.1	1101.3	1096.2	-5.1	205.5	204.1	-1.4	13.65	13.65	0	14.21	14.21	0	14.89	14.89	0
11	46	tonsil	TXN2M0	12.2	12.9	0.7	826.9	830.5	3.6	97.3	98.4	1.1	15.37	15.37	0	15.11	15.11	0	16.65	16.65	0
Averages	59			22.8	23.3	0.5	691.9	682.6	-9.2	126.4	126.8	0.4	13.4	13.4	0.0	13.1	13.1	0.0	14.4	14.2	-0.2

The GTV as measured by CT before treatment and the GTV measured by CBCT mid-treatment reveals that tumor volume increased for eight patients, one patient had no change in GTV, and two patients had a decrease in GTV (Table 1). These differences were statistically significant (p=0.0015).

The planning target volumes (PTV1 and PTV2) were measured similarly before and during treatment (Table 1). Five patients had a change in volume between 1% and 5% and six patients had a change in a volume greater than 5% for PTV1. Changes for PTV2 were less than 2% for all patients. No statistically significant difference was observed for each patient's measured volumes.

Neck diameter did not change for all patients except two, where the lower neck diameter decreased from 12.91 to 12.51 cm and from 16.72 to 15.44 cm (Table 1).

Displacements of the center of mass between the two tumor images were measured (Table 2) with all displacements less than 0.1 cm for all but one patient. Coefficients of similarity (Table 3) determined for the images were 0.93 or greater.

**Table 2:** Measured displacement of the tumoral center of mass for PTV1 and PTV2.

#	PTV1, cm				PTV2, cm			
	x	y	z	distance	x	y	z	distance
1	0	0	0.01	0.01	0.01	-0.01	0.01	0.02
2	0.01	0	0.02	0.02	0	0	0.04	0.04
3	0	0	-0.01	0.01	0	0	-0.03	0.03
4	-0.05	0	0.62	0.62	0	0	-0.04	0.04
5	0	0	0.03	0.03	0	-0.01	-0.01	0.01
6	0	-0.01	-0.01	0.01	-0.01	-0.01	-0.03	0.03

**Table 3:** Coefficients of similarity for PTV1 and PTV2 shapes using images taken before and during treatment.

#	PTV1 Coefficient of similarity	PTV2 Coefficient of similarity
1	0.93	0.96
2	0.97	0.97
3	0.97	0.94
4	0.94	0.94
5	0.97	0.97
6	0.97	0.95
7	0.97	0.96
8	0.97	0.96
9	0.95	0.97
10	0.98	0.97
11	0.97	0.96

## Discussion

In this study, we followed nine male and two female patients diagnosed with SCCHN through radiation treatment. The sex proportion of the patients is reflected in other studies and worldwide epidemiological trends [15,16].

In Peru, head and neck cancers are diagnosed in advanced clinical stages, due to the lack of early diagnosis and screening centers nationwide. This is compounded by the fact that even advanced cases have mild symptoms, as evidenced by the low ECOG scores of the patients at admission. In our study, 82% of patients were found in clinical stage III and IV. Tumor staging is a well-defined prognostic factor for head and neck cancers, and studies report relapses in about 50% of cases with locally advanced tumors [16].

SCCHN is treated by several modalities, including radiation, surgery, and chemotherapy. Newer approaches such as immunotherapy [17], peptide vaccines [18], nutritional management [19], pain management [20], and even adjuvant cannabis therapy [21] have been investigated to improve patient outcomes. Surgery, radiotherapy, and chemotherapy can cause swelling and displacement of anatomical structures around the treatment site, which may require changes in the radiotherapy treatment plan to achieve a good result [22,23].

The only statistically significant difference in the treatment group was for an increase in GTV, likely due to radiation-induced swelling. An average increase of 0.5 ± 0.4 cm^3^ was observed. This difference was not clinically significant for most patients and no RT re-planning was ordered due to an increase in tumor volume. Edema is a well-known side effect of neck irradiation and is likely due to the destruction of endothelial cells of blood vessels. It frequently occurs during and following radiotherapy [23].

No statistically significant differences were observed for PTV1, PTV2, and neck measurements, although some patients did have changes. Tumor displacement among all patients was small and image coefficients of similarity were very high. As a group, this information shows that only small changes were observed during VMAT. However, measuring patients as a group might not be the only valid way of interpreting these results.

Taken individually, some patients had measurable changes in some parameters, while others did not change; the pattern of anatomical changes was different between patients (Tables 1, 2). For instance, patients 1 and 5 had a measurable change to lower neck diameter, while others did not. Likewise, the GTV decreased for patient 10, but stayed nearly the same or increased in all others. Patient 5 saw some changes to tumor position, while most others did not. During treatment, only one image was taken, so it was not possible to observe trends or transient changes in anatomy or patient positioning.

Therefore, making broad recommendations as to what criteria a patient must have to benefit from ART and re-planning has proven difficult. It has been estimated that only 10%-20% SCCHN patients require re-planning after mid-treatment imaging [24,25]. A separate study found that positional deviation that remains after correcting the actual positioning to the planned position was observed to be greater than 5 mm in 10% of patients if imaging is performed daily, and in 33% of patients submitted to weekly imaging [8]. Different factors such as weight loss, disease progression, the timing of the second image, location of the primary tumor, nutritional intervention, functional impairment, immobilization-related factors, shrinkage or deformation of the treatment volume, displacement of the lymph nodes and medial parotid gland and type of treatment have all been evaluated as triggers for re-planning [8,25,26]. Other studies report more frequent changes to weight, neck, OAR, and tumor shape and position than those observed here. The dose impact of these changes has been shown to range from insignificant to variations on the order of 2-3 Gy when compared to the plan [26].

None of the discussed criteria were observed in the patients in the study, and the thermoplastic immobilization devices used maintained a good fit throughout the treatment, making replanning unnecessary. This might mean that radiotherapy replanning is not needed as frequently as suggested, but it could also be an artifact of infrequent patient imaging, which makes it difficult to observe transient changes and trends in anatomy during the treatment. Furthermore, due to hospital limitations, a CBCT follow-up image rather than a CT image was taken. This was a lower-quality image. 

## Conclusions

These results shed light on a complex problem that includes intersecting needs of the patient, oncologist, and hospital. Although IRGT/VMAT apparently holds the promise to improve treatment outcomes, replanning radiotherapy did not seem necessary for the patients in the study, as evidenced by slight to no change in anatomical features, GTV, PTV1, and PTV2, as well as a high degree of similarity between images taken pre- and mid-treatment.

Alternatively, only one image was taken during treatment, and it is likely that more images would have better elucidated trends in tumor displacement and volume as well as anatomical changes. Therefore, the results may also suggest that frequent imaging is necessary for IGRT/VMAT to be effective. This presents challenges in public hospitals in developing countries with high patient demand for medical equipment and a lack of trained technicians. Therefore, clearer criteria for patients at risk for replanning would be most helpful in improving patient outcomes and prioritizing the efficient use of equipment and specialist time.

## References

[1] Torre LA, Bray F, Siegel RL, Ferlay J, Lortet-Tieulent J, Jemal A (2015). Global cancer statistics, 2012. CA Cancer J Clin.

[2] Marur S, Forastiere AA (2016). Head and neck squamous cell carcinoma: update on epidemiology, diagnosis, and treatment. Mayo Clin Proc.

[3] Jung K, Narwal M, Min SY, Keam B, Kang H (2020). Squamous cell carcinoma of head and neck: what internists should know. Korean J Intern Med.

[4] Robar JL, Day A, Clancey J (2007). Spatial and dosimetric variability of organs at risk in head-and-neck intensity-modulated radiotherapy. Int J Radiat Oncol Biol Phys.

[5] Ahn PH, Chen CC, Ahn AI (2011). Adaptive planning in intensity-modulated radiation therapy for head and neck cancers: single-institution experience and clinical implications. Int J Radiat Oncol Biol Phys.

[6] Lee C, Langen KM, Lu W (2008). Evaluation of geometric changes of parotid glands during head and neck cancer radiotherapy using daily MVCT and automatic deformable registration. Radiother Oncol.

[7] Beltran M, Ramos M, Rovira JJ (2012). Dose variations in tumor volumes and organs at risk during IMRT for head-and-neck cancer. J Appl Clin Med Phys.

[8] Romero P, Villafranca M, Rico A, Manterola MT, Vila M, Domínguez MA (2009). Radioterapia guiada por imagen. Impacto clínico. Anales del Sistema Sanitario de Navarra.

[9] Nguyen D, Lyu Q, Ruan D, O'Connor D, Low DA, Sheng K (2016). A comprehensive formulation for volumetric modulated arc therapy planning. Med Phys.

[10] Kearney M, Coffey M, Leong A (2020). A review of image guided radiation therapy in head and neck cancer from 2009-2019 - best practice recommendations for RTTs in the clinic. Tech Innov Patient Support Radiat Oncol.

[11] Chu KP, Le QT (2011). Intensity-modulated and image-guided radiation therapy for head and neck cancers. Front Radiat Ther Oncol.

[12] Perdomo S, Martin Roa G, Brennan P, Forman D, Sierra MS (2016). Head and neck cancer burden and preventive measures in Central and South America. Cancer Epidemiol.

[13] Fagan JJ, Noronha V, Graboyes EM (2021). Making the best of limited resources: improving outcomes in head and neck cancer. Am Soc Clin Oncol Educ Book.

[14] International Commission on Radiation Units and Measurements (1999). ICRU report 62: prescribing, recording, and reporting photon beam therapy (supplement to ICRU report 50). https://www.icru.org/report/prescribing-recording-and-reporting-photon-beam-therapy-report-62/.

[15] Cook MB, Dawsey SM, Freedman ND (2009). Sex disparities in cancer incidence by period and age. Cancer Epidemiol Biomarkers Prev.

[16] Cadoni G, Giraldi L, Petrelli L (2017). Prognostic factors in head and neck cancer: a 10-year retrospective analysis in a single-institution in Italy. Acta Otorhinolaryngol Ital.

[17] Yokota T, Homma A, Kiyota N (2020). Immunotherapy for squamous cell carcinoma of the head and neck. Jpn J Clin Oncol.

[18] Sun Z, Sun X, Chen Z, Du J, Wu Y (2022). Head and neck squamous cell carcinoma: risk factors, molecular alterations, immunology and peptide vaccines. Int J Pept Res Ther.

[19] Curtin P, Akbar A, Kramer H, Iqbal A, Markossian T (2020). The status of nutritional management guidelines for head and neck cancer patients. Cureus.

[20] Mirabile A, Airoldi M, Ripamonti C (2016). Pain management in head and neck cancer patients undergoing chemo-radiotherapy: clinical practical recommendations. Crit Rev Oncol Hematol.

[21] Caputo MP, Rodriguez CS, Padhya TA, Mifsud MJ (2021). Medical cannabis as adjunctive therapy for head and neck cancer patients. Cureus.

[22] Mali SB (2016). Adaptive radiotherapy for head neck cancer. J Maxillofac Oral Surg.

[23] Glastonbury CM, Parker EE, Hoang JK (2010). The postradiation neck: evaluating response to treatment and recognizing complications. AJR Am J Roentgenol.

[24] Chen AM, Daly ME, Cui J, Mathai M, Benedict S, Purdy JA (2014). Clinical outcomes among patients with head and neck cancer treated by intensity-modulated radiotherapy with and without adaptive replanning. Head Neck.

[25] Figen M, Çolpan Öksüz D, Duman E (2020). Radiotherapy for head and neck cancer: evaluation of triggered adaptive replanning in routine practice. Front Oncol.

[26] García-Mollá R, Rubio PS, Alandí JB, Herrera MAC, Valverde FL (2021). Implementación y uso clínico de la radioterapia adaptativa. Informe del grupo de trabajo de radioterapia adaptativa de la Sociedad Española de Física Médica (SEFM). Rev Fis Med.

